# Supplementary material for: Enhancing Acetate Utilization in Phaeodactylum tricornutum through the Introduction of Acetate Transport Protein
Source: Biomolecules. 2024 Jul 9;14(7):822. doi: 10.3390/biom14070822 (PMC11274376; doi:10.3390/biom14070822)
Supplement: Supplementary file 1 [file biomolecules-14-00822-s001.zip › Enhancing Acetate Utilization in Phaeodactylum tricornutum-Supplementary Materials.pdf]

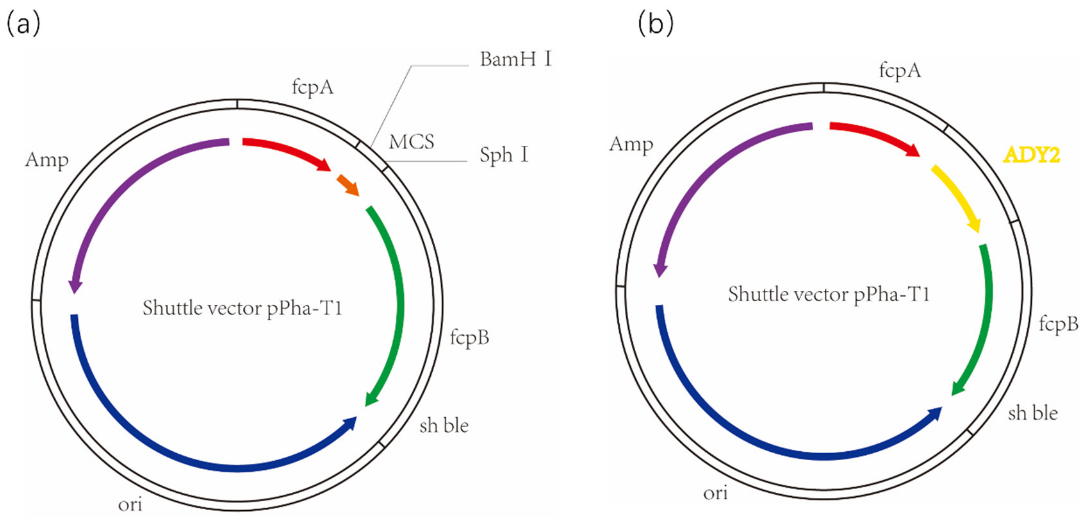

Figure S1. Construction of the recombinant vector pPha-T1-ADY2.

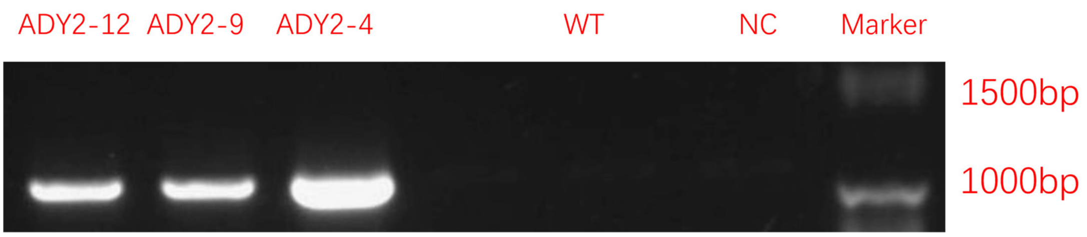

Figure S2. Positive confirmation of pPha-T1-ADY2 transformants. WT, wild-type strain; NC, negative control.

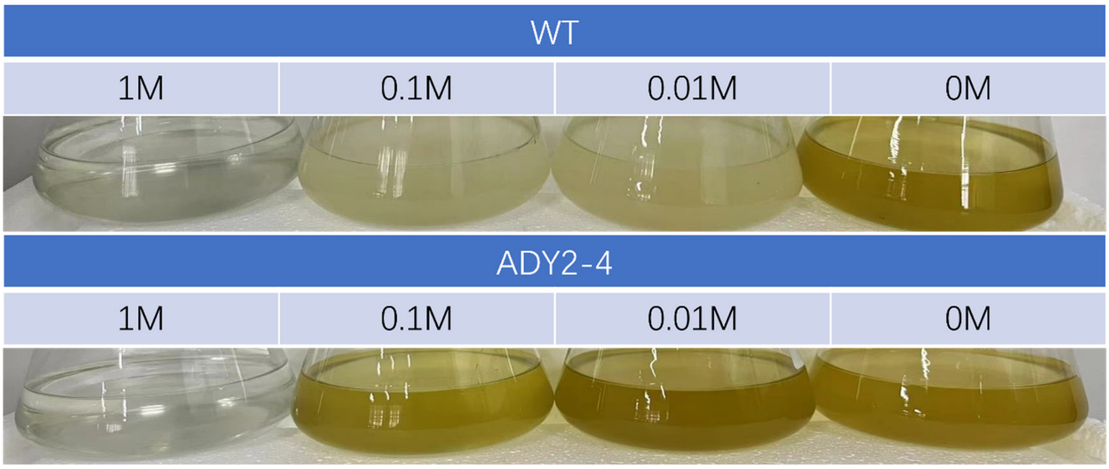

Figure S3. The growth comparison of the wild-type and mutant strain ADY2-4 under different concentrations of sodium acetate after 7 days.

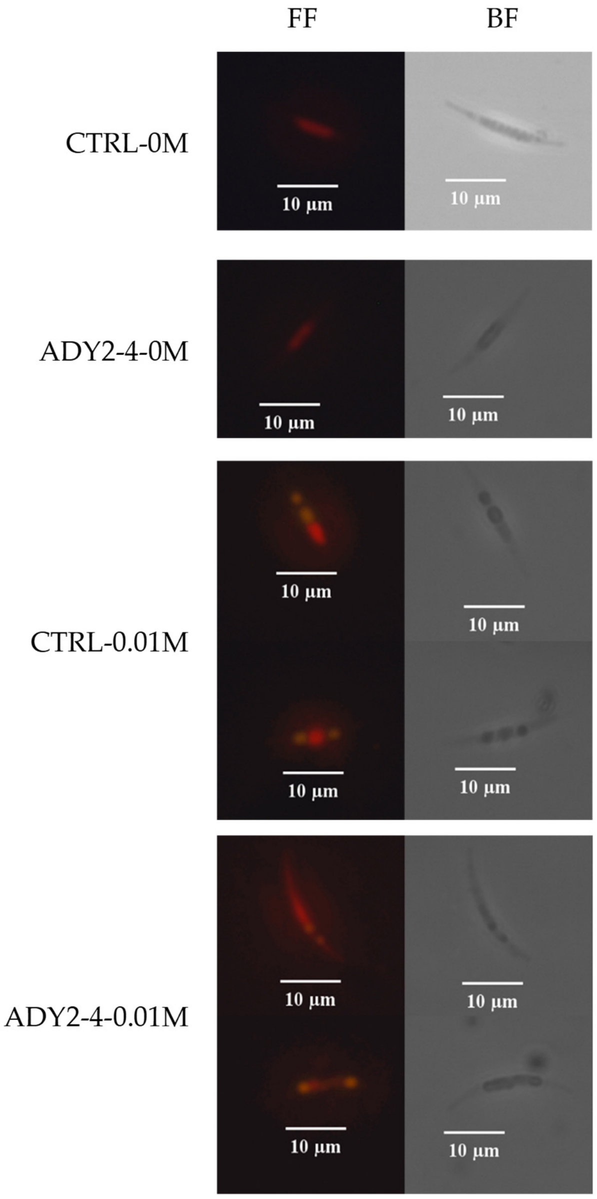

Figure S4. The cell morphology comparison of the wild-type and mutant strain ADY2-4 in the late-exponential growth phase under conditions with and without the addition of NaAc. FF, fluorescence filed, stained with Nile Red; BF, bright field.
