# Supplementary material for: Enhancing Acetate Utilization in Phaeodactylum tricornutum through the Introduction of Acetate Transport Protein
Source: Biomolecules. 2024 Jul 9;14(7):822. doi: 10.3390/biom14070822 (PMC11274376; doi:10.3390/biom14070822)
Supplement: Supplementary file 1 [file biomolecules-14-00822-s001.zip › instruction.pdf]

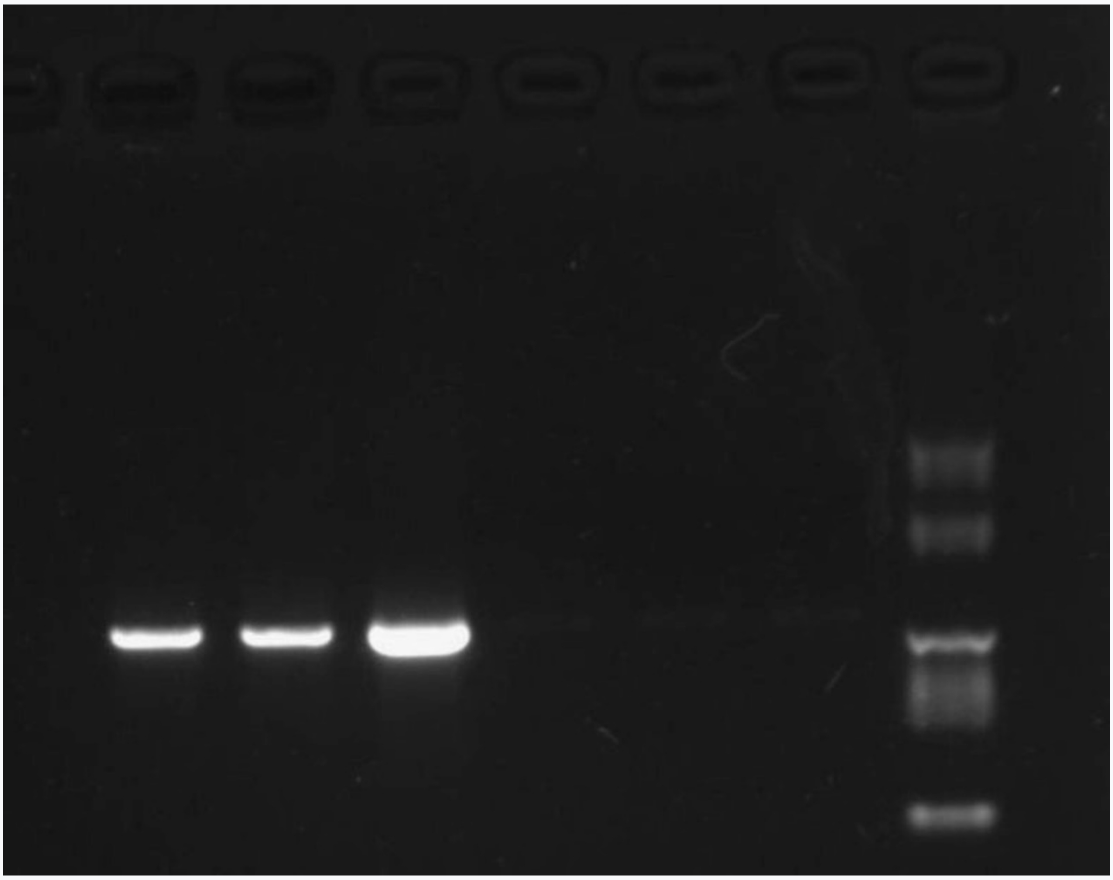

This figure corresponds to Figure. S2 in the supplementary material.

From left to right, each lane corresponds to: ADY2-12, ADY2-9, ADY2-4, WT, negative control and 2000bp marker
